# Effects of Electroacupuncture on Chronic Unpredictable Mild Stress Rats Depression-Like Behavior and Expression of p-ERK/ERK and p-P38/P38

**DOI:** 10.1155/2015/650729

**Published:** 2015-08-20

**Authors:** Jian Xu, Yanling She, Ning Su, Ruixin Zhang, Lixing Lao, Shifen Xu

**Affiliations:** ^1^Shanghai Municipal Hospital of Traditional Chinese Medicine, Shanghai University of Traditional Chinese Medicine, Shanghai 200071, China; ^2^Guangzhou University of Traditional Chinese Medicine, Guangzhou 510405, China; ^3^University of Maryland School of Medicine, Baltimore, MD 21201, USA; ^4^School of Chinese Medicine, The University of Hong Kong, Pokfulam, Hong Kong

## Abstract

We investigate the antidepressant-like effect and mechanism of electroacupuncture (EA) on a chronic unpredictable mild stress rats depression-like behavior. In our study, depression in rats was induced by unpredictable chronic mild stress (UCMS) and isolation for four weeks. Male Sprague-Dawley rats were randomly divided into four groups: Normal, Model, EA, and Sham EA. EA treatment was administered for two weeks, once a day for five days a week. Two acupoints, Yintang (EX-HN3) and Baihui (GV20), were selected. For sham EA, acupuncture needles were inserted shallowly into the acupoints: EX-HN3 and GV20. No electrostimulator was connected. The antidepressant-like effect of the electroacupuncture treatment was measured by sucrose intake test, open field test, and forced swimming test in rats. The protein levels of phosphorylated extracellular regulated protein kinases (p-ERK1/2)/ERK1/2 and p-P38/P38 in the hippocampus (HP) were examined by Western blot analysis. Our data demonstrate that EA treatment decreased the immobility time of forced swimming test and improved the sucrose solution intake in comparison to unpredictable chronic mild stress and placebo sham control. Electroacupuncture may act on depression by enhancing p-ERK1/2 and p-p38 in the hippocampus.

## 1. Introduction

Depression, a debilitating illness that exerts a large emotional and monetary cost on society [1], involves emotion, cognition, and physical symptoms. Clinical depression is characterized in the DSM-IV-R (APA2004, DSM IV-R, Masson: APA) by low mood (sadness, loss of motivation, feelings of worthlessness/guilt, and suicidal ideas), reduced cognition (concentration/attention deficit), low or impaired psychomotor activity, fatigue, loss of appetite, and sleeplessness. The illness is characterized by loss of confidence in oneself, the world, and the future, and it is often preceded by a period of acute or chronic stress [2]. Depression is a serious condition that often requires medical intervention. Although clinically it is treated with antidepressants, however, not all patients respond to antidepressant treatment. Moreover, the therapeutic effect takes several weeks to manifest and is often accompanied by unwanted side effects [3]. For these reasons, new strategies for treating depression are urgently needed.

Acupuncture is a major therapy that has been used in China for thousands of years to treat various medical and psychiatric conditions [4], and some studies substantiate its benefits for depression. Duan et al. reported that somatic symptoms of depression were alleviated faster and with fewer adverse effects when electroacupuncture (EA) with Baihui (GV20) and Yintang (EX-HN3) was combined with the antidepressant fluoxetine [5]. Manber et al. recently reported that acupuncture was more effective than nonspecific sham acupuncture control on depression in pregnant women and that the response rate was comparable to that observed for conventional treatments of similar length [6]. A review shows that acupuncture therapy is safe and effective in depressive disorder and poststroke depression and may be considered an alternative option for treating both disorders [7].

It has been reported that acupuncture treatment could activate ERK-CREB pathway and alleviate depressive-like behavior [8]. The mitogen-activated protein kinases (MAPKs) in mammals include c-Jun NH2-terminal kinase (JNK), p38MAPK, and extracellular signal-regulated kinase (ERK). These enzymes are serine-threonine protein kinases that regulate various cellular activities including proliferation, differentiation, apoptosis or survival, inflammation, and innate immunity. The compromised MAPK signaling pathways contribute to the pathology of diverse human diseases including neurodegenerative disorders and cognitive disorder [9]. ERK1/2 and p38MAPKs are members of the MAPK family that are activated by a variety of environmental stresses and inflammatory cytokines. Currently available data suggest that ERK- and p38-responsive MAPKs function during mitogenic and stressful conditions, respectively [10].

In the present study, we used sucrose intake test, open field test, and forced swimming test to determine whether EA affects UCMS-induced depression-like behavior. To examine the mechanism underlying the effect of EA on UCMS-induced depressive-like hippocampal nerve system responses, we used Western blot analysis to examine the protein levels of p-ERK1/2/ERK1/2 and p-P38/P38 in the hippocampus.

## 2. Materials and Methods

### 2.1. Animal Preparation

Male Sprague-Dawley rats weighing between 180 and 200 grams were obtained from the Medical School of Shanghai Jiaotong University, Shanghai, China. They were kept under controlled environmental conditions (22 ± 1°C, relative humidity 40–60%, alternate dark-light cycles from 9:00 a.m. to 9:00 p.m., and water and food* ad libitum*). The rats were housed separately in standard cages and given one week to adapt to the environment before experimentation. The animal protocols were approved by the Ethical Committee of Shanghai Jiaotong University of Medical Sciences.

### 2.2. Experiment Design

The rats were randomly divided into four groups of 7 rats in each group: Normal group, Model group, EA group, and Sham EA group. The Normal group was given no stress except general handling for 4 weeks. The rats in the Model group, EA group, and Sham group were exposed to CUMS twice a day for 2 weeks. Afterwards, the Model group was sequentially exposed to CUMS twice a day for 2 weeks; the EA group received EA treatment in the morning and CUMS for two weeks, once a day in the afternoon; the Sham EA group received EA treatment in the morning and CUMS once a day in the afternoon for two weeks. We employed the three commonly used behavioral tests for depression: the sucrose intake test, forced swim tests (FST), and open field tests (e.g., (a) total distance traveled, (b) distance traveled within a central area, (c) time spent in central area, and (d) rearing and grooming). The tests were conducted for four weeks after the initial intervention. After the behavioral tests had been completed, rats were sacrificed by decapitation for Western blot analysis.

### 2.3. Chronic Unpredictable Stress

The CUS paradigm was performed as previously described [11] with minor modifications. Rats that are 8 weeks old were randomly exposed to one of the following stressors: food deprivation, water deprivation, cage tilt 45, swimming in 10°C ice water, restraint stress, cage shaking, and light on or light off twice daily for two weeks. Subsequently, according to different groups, they were exposed twice or once daily for another two weeks. Stressors were given randomly at different times of the day to establish unpredictability. The schedule of stressors is shown in Table 1. The rats in the Normal control group were housed undisturbedly except for necessary procedures such as routine cage cleaning.

### 2.4. Acupuncture Treatment Procedures

We used two acupoints, Yintang (EX-HN3) and Baihui (GV20), commonly employed in the clinic for the treatment of depression [12, 13]. In humans, EX-HN3 is located midway between the medial ends of the two eyebrows and GV20 on the midline at the apex of the skull. Anatomical equivalents of these points were located in the animals [10]. After the skin had been cleaned with alcohol swabs, disposable acupuncture needles (0.25 × 13 mm, Suzhou Medical Appliance Factory, Suzhou, China) were inserted about 5 mm deep into the acupoints. An electrostimulator, Huatuo's Acupoint Nerve Stimulator (SDZ-V, Huatuo Medical Technology Co., Ltd., Suzhou, China), was connected, and the electrical current was delivered to the needles. The anode was inserted into EX-HN3; the cathode was inserted into GV20. EA frequency was held constant at 2 Hz, pulse width 0.3 ms, and intensity was adjusted slowly over the period of approximately two minutes to the designated level (i.e., 1, 2, or 3 mA). Mild muscle twitching was observed. The needles were retained for fifteen minutes. As previously reported by Lao et al., each rat was placed in an inverted clear 5′′ × 8′′ × 11′′ plastic chamber during the treatment and was neither restrained nor given anesthetic [14]. The animals remained awake and still during treatment, and no signs of distress were observed. Treatment was given for two weeks once a day at approximately 10:00 a.m., from Monday to Friday.

### 2.5. Sham Control Procedure

For control, after the skin was cleaned with alcohol swabs, disposable acupuncture needles (0.25 × 13 mm, Suzhou Medical Appliance Factory, Suzhou, China) were inserted about 2 mm deep into the acupoints: EX-HN3 and GV20. A piece of adhesive tape was secured at the surface. No electrostimulator was connected. The needles were retained for fifteen minutes. Each rat was placed in an inverted clear 5′′ × 8′′ × 11′′ plastic chamber during the treatment and was neither restrained nor given anesthetic too. Control and treatment animals were treated on the same schedule to make the procedures comparable.

### 2.6. Behavioral Tests

**Figure 1:**
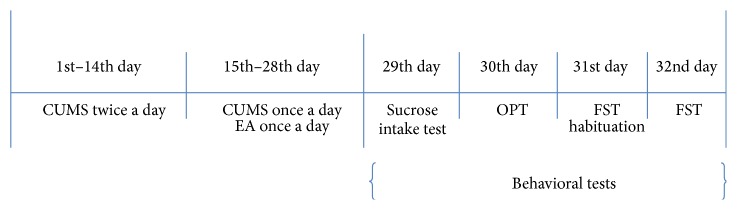
Timeline for all procedures.

Behavioral tests were performed four weeks after the initial intervening. They were conducted between 4 p.m. and 9 p.m. and videotaped in a random order for later scoring by two observers blinded to the treatment assignment. Interrater reliability correlation was >0.90 for all tests. Timeline for all procedures can be seen in Figure 1.

#### 2.6.1. FST [15]

The FST was conducted over three days and consisted of two pretests and a test. During the pretests, which were for training purposes, each rat was placed for 15 minutes in a clear Plexiglas cylinder (25 cm in diameter, 65 cm high) filled with 50 cm of 25 ± 1°C water. The results of the pretests were not recorded. Twenty-four hours after the second pretest, each rat was returned to the apparatus for five minutes. Upon completion of the trial, the animal was dried thoroughly and placed back in its home cage. The apparatus was drained and cleaned after each use. Behavioral changes were scored for analysis following the methods previously reported [16]: immobility was defined as making only those movements necessary to stay afloat; longer immobility indicated more serious depression. Immobility latency was operationally defined as at least ten consecutive seconds of immobility.

#### 2.6.2. OFT [17]

The open field test (OFT) is a commonly used qualitative and quantitative measure of general locomotor activity and willingness to explore in rodents [18]. The open field is a table that may have surrounding walls to prevent escape. Usually, the field is marked in a grid and square crossings, rearing, and time spent moving are used to assess the activity of the rodent. In modern open field apparatus, infrared beams can be used to automate the assessment process. The open field test was designed to measure anxiety and depression, as well as behavioral responses such as locomotive activity and exploratory behaviors. Some studies used the open field test to measure depression [19, 20]. Rats in the open field were videomonitored with an automated activity monitoring system (TRU Scan, CoulBourn Instruments, USA). The rat was placed in a randomized starting corner of a square black apparatus (50 cm long × 50 cm wide) with walls 100 cm high. Its behavior, including total distance traveled, total movement time, distance traveled within the central area, and time spent in the central area, was recorded automatically for five minutes and analyzed with computer software. Incidents of rearing and grooming (face washing, body and genital grooming, body and paw licking, and scratching) were counted by an investigator. Total distance traveled indicates an animal's spontaneous activity. Time spent and distance traveled within the central area show the animal's ability to explore. Rearing and grooming incidents indicate the animal's curiosity. All activity was recorded by a video camera mounted above the open field and scored in real time (or digitized and scored later) by an advanced motion recognition software package that detects and analyzes movements.

#### 2.6.3. Sucrose Intake Test

The sucrose intake test is a behavioral task used to assess the degree of anhedonia in rats [21]. The sucrose intake test was performed on the first day of the test. Before the test, the rats were habituated for 24 hours to two bottles: one with 1% sucrose (Sigma) and the other with tap water. On the last day of EA treatment, all rats were deprived of water for 24 h. Then the rats were given 24 h exposure to the two identical bottles again to test for fluid consumption. Two-bottle tests for each cage were adopted throughout the procedure. Sucrose solution consumption was recorded by calculating the volume of test solution.

#### 2.6.4. Western Blot Analysis

After the behavioral tests had been completed, rats were sacrificed by decapitation for Western blot analysis. Then the HP was dissected. Brain tissue was homogenized in an extraction buffer containing SDS lysis buffer, protease inhibitor mixture, and phosphatase inhibitor mixture. The homogenates were centrifuged at 13,000 rpm for 15 min at 4°C. The same level of total proteins was loaded on the gels and separated on 10% SDS-PAGE and transferred onto PVDF membranes. Blots were blocked in blocking buffer (1*∗*TBS, 0.1% Tween-20 with 5% nonfat dry milk) for 1 hour at room temperature. Following washing, blots were incubated overnight at 4°C with primary antibodies against ERK1/2 (1 : 1000), ERK1/2 phosphorylated on Thr202/Tyr204 (1 : 1000), p38MAPK (1 : 1000), and p38MAPK phosphorylated on Thr180/Tyr182 (1 : 1000). After washing 2 times in TBS-T and 3 times in 1% milk-TBST for 5 min each, blots were incubated with goat anti-mouse or anti-rabbit secondary antibody for 1 h at room temperature and then washed 2 times in 1% milk-TBST and 3 times in TBS-T for 5 minutes each. Band intensity was detected by LI-COR Odyssey infrared fluorescence scanning imaging system and quantified by ImageJ. The relative level of each signal protein was calculated as the ratio between phosphorylated and total protein [22–24].

### 2.7. Statistical Analysis

Data were analyzed using SPSS15.0 for Windows XP. All data were expressed as mean ± SD. An analysis was performed by ANOVA, with Bonferroni's test for multiple comparisons, and by* t*-test; a *P* value of <0.05 was considered statistically significant.

## 3. Results

### 3.1. FST

**Figure 2:**
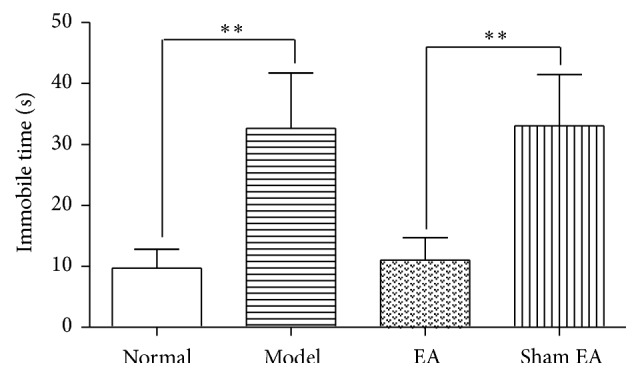
Immobility time (in seconds) of forced swimming test in the following groups (*n* = 7 per group): Normal, Model, EA, and Sham EA. ^*∗∗*^ *P* < 0.01.

The forced swimming results are shown in Figure 2. There are significant differences observed among groups [*F*_(3,24)_ = 26.93, *P* < 0.01]. The Model rats showed a significant increase in the immobile time compared to Normal rat (*P* < 0.01). EA treatment had a significant effect on the decreased immobile time compared to sham control (*P* < 0.01), suggesting that EA may decrease immobile time of FST in depressed rat, but Sham EA had no effect on it.

### 3.2. Open Field Tests

**Figure 3:**
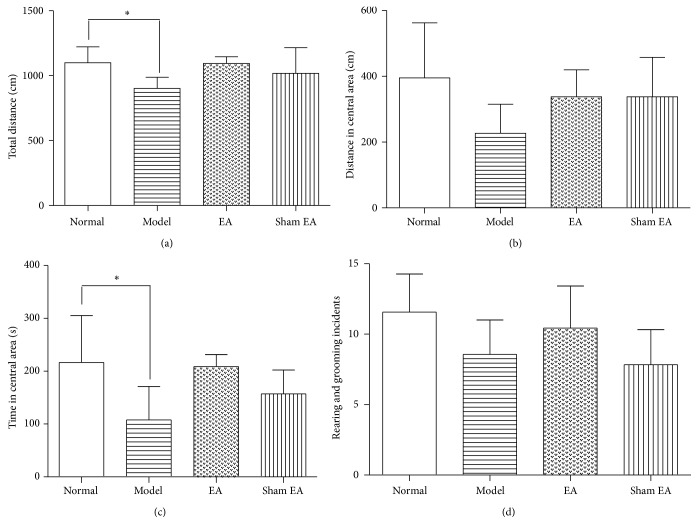
Open field test scores in the following groups (*n* = 7 per group): Normal, Model, EA, and Sham EA. (a) Total distance, (b) distance in the central area, (c) time in central area, and (d) rearing and grooming incidents. ^*∗*^ *P* < 0.05. There was no significant difference between EA and Sham EA in all parameters of OFT.

The results of the open field tests are shown in Figure 3. In the total distance, there was a significant difference among groups [*F*_(3,24)_ = 3.965, *P* < 0.05]. The Model rats showed a significant decrease of the total distance compared to Normal rat (*P* < 0.05). However, there was no significant difference between EA and sham control (*P* > 0.05). In the central distance, there was no significant difference among groups [*F*_(3,24)_ = 2.442, *P* > 0.05]. For time in the central area, there was a significant difference among groups [*F*_(3,24)_ = 4.812, *P* < 0.01]. The Model rats showed a significant decrease of time in the central area compared to Normal rat (*P* < 0.05). EA treatment had no significant effect on the time in the central area compared to sham control (*P* > 0.05). There was no significant difference observed among groups in rearing and grooming incidents [*F*_(3,24)_ = 2.71, *P* > 0.05], suggesting that EA had no significant effect on open field tests compared to sham control.

### 3.3. Sucrose Intake Test

**Figure 4:**
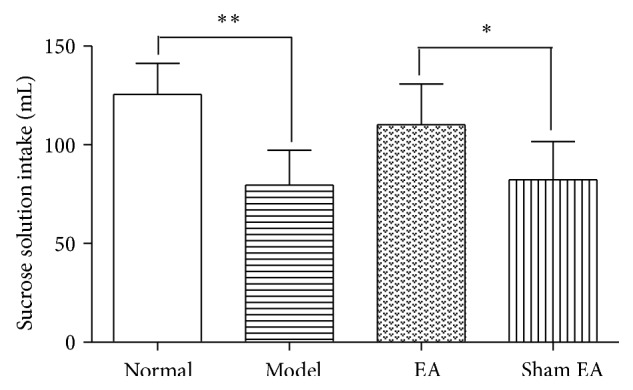
Sucrose solution intake in the following groups (*n* = 7 per group): Normal, Model, EA, and Sham EA. ^*∗*^ *P* < 0.05, ^*∗∗*^ *P* < 0.01.

The results of the sucrose intake test are shown in Figure 4. The sucrose solution intake significantly differed among groups [*F*_(3,24)_ = 10.19, *P* < 0.01]. The sucrose intake was significantly reduced in model rat compared to Normal group (*P* < 0.01). It was improved after EA treatment compared to sham control (*P* < 0.05), suggesting that EA may improve sucrose solution intake in depression rat. However, Sham EA had no effect on it.

### 3.4. The Effects of Western Blot Analysis

#### 3.4.1. The Effect of p-ERK1/2 Western Blot Analysis

**Figure 5:**
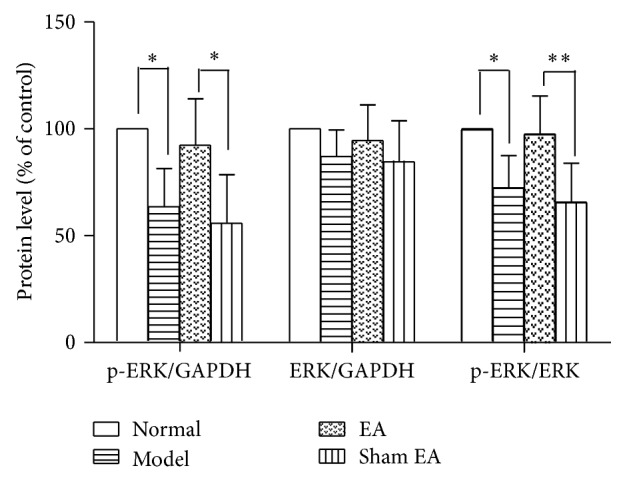
ERK and p-ERK protein expression in the hippocampus in the following groups (*n* = 6 per group): Normal, Model, EA, and Sham EA. ^*∗*^ *P* < 0.05, ^*∗∗*^ *P* < 0.01.

**Figure 6:**
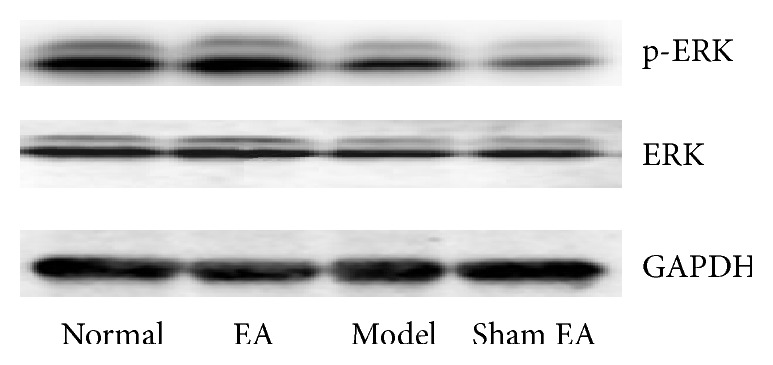
Representative Western blots showing levels of p-ERK, ERK in the hippocampus of the following groups (*n* = 6 per group): Normal, Model, EA, and Sham EA.

Western blot analysis revealed that there was no significant difference in the ERK1/2 protein level among groups in the HP [*F*_(3,20)_ = 1.499, *P* > 0.05]. However, a p-ERK1/2 level significantly differed among groups in the HP [*F*_(3,20)_ = 8.408, *P* < 0.01]. Chronic stress significantly decreased p-ERK1/2 in the HP (*P* < 0.05) compared to the Normal group. EA treatment significantly increased p-ERK1/2 level in HP compared to Sham EA (*P* < 0.05). At the same time, from the ratio between p-ERK1/2 and ERK, there was a significant difference among groups [*F*_(3,20)_ = 8.284, *P* < 0.01]. Model group significantly decreased compared to Normal group (*P* < 0.05); EA treatment increased the ratio between p-ERK1/2 and ERK compared to sham control (*P* < 0.01), suggesting that EA may reverse the deficits in p-ERK in the HP on depression rats. However, sham control had no effect on it (see Figures 5 and 6).

#### 3.4.2. The Effect of p-p38 Western Blot Analysis

**Figure 7:**
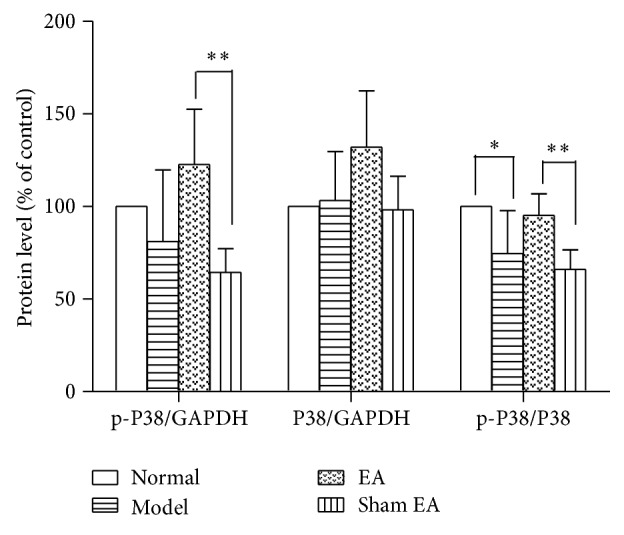
P38 and p-P38 protein expression in the hippocampus in the following groups (*n* = 6 per group): Normal, Model, EA, and Sham EA. ^*∗*^ *P* < 0.05, ^*∗∗*^ *P* < 0.01.

**Figure 8:**
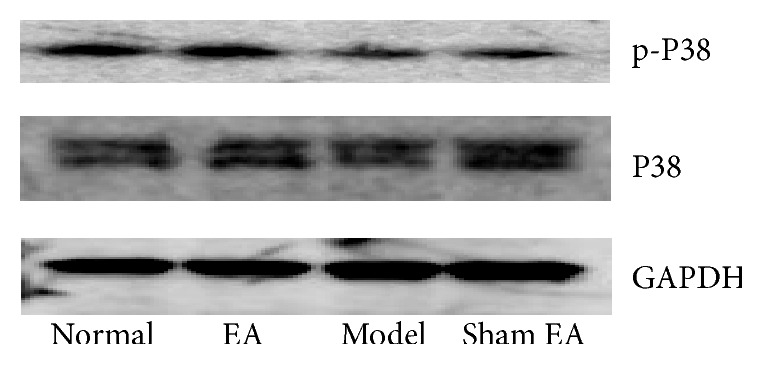
Representative Western blots showing levels of p-P38, P38 in the hippocampus of the following groups (*n* = 6 per group): Normal, Model, EA, and Sham EA.

Western blot analysis revealed that there was no significant difference in the p38 protein level among groups in the HP [*F*_(3,20)_ = 3.104, *P* > 0.05]. However, a p-p38 level significantly differed among groups in the HP [*F*_(3,20)_ = 5.895, *P* < 0.01]. Unpredictable chronic mild stress decreased p-p38 in the HP compared to the Normal rat. EA treatment significantly increased p-p38 level in HP compared to sham control (*P* < 0.01). At the same time, from the ratio between p-p38 and p38, there were significant differences among groups [*F*_(3,20)_ = 8.102, *P* < 0.01]. Model group decreased the ratio significantly compared to Normal group (*P* < 0.01). EA treatment had a significant effect on depression model rats compared to sham control (*P* < 0.01) suggesting that EA may reverse the deficits in p-p38 in the HP on depression rats. However, Sham EA had no effect on it (see Figures 7 and 8).

## 4. Discussion

The present study showed that EA treatment decreased the immobility time of forced swimming test and improved the sucrose solution intake in comparison to placebo sham control (*P* < 0.05, *P* < 0.01). However, for the open field test, EA had no significant effect compared to placebo sham control. These results suggest that EA stimulation may alleviate some symptoms in this animal model of depression. Some other studies that researched antidepressant drugs showed that the results in OFT also had no significant difference [25, 26]. For example, fluoxetine decreased immobility of forced swimming test but did not affect activities in an open field [25]. Baicalein reduced the immobility time in the forced swimming test and tail suspending test of mice, but there was no effect on open field test [27]. Our results are similar to those reported by others [28–30]. Lee et al. [28] observed the effect of acupuncture in a rat model of depression. They found that acupuncture treatment showed significantly less immobility time compared to control. Neither open field tests nor MWMs were performed. Dos Santos Jr. et al. [29] used rats to investigate the effects of EA on depressive-like symptoms with a learned helplessness test and the FST. The EA group showed significantly enhanced active avoidance in the learned helplessness test and less immobility in the FST (*P* < 0.01) compared to sham control, but with EA there was no effect on number of squares crossed in an open field test. We suppose that maybe open field test is not sensitive enough to depression.

We also found that chronic stress exposure caused deficits in p-ERK and p-p38 in the HP, which could be reversed by electroacupuncture treatment. There was a significant difference between EA treatment and sham control (*P* < 0.01). ERK and p38 belong to MARK family. ERK pathway in an intracellular signaling cascade is implicated in several forms of learning, memory, and neuroplasticity [31]. Stress caused a reduction in ERK phosphorylation in both the hippocampus and the prefrontal cortex, whereas it led to a nonsignificant decrease in BDNF levels only in the HP. The involvement of ERK activation in the stress response and antidepressant therapy has also been shown in another study [32]. The p38MAPK mainly functioned as mediators of cellular stresses since increasing evidence implicates stress as an important factor in the vulnerability to depression [33].

The mechanisms of acupuncture on depression have been explored. We previously reported that acupuncture may act on depression by mediating the regulation of central monoamine neurotransmitters, including norepinephrine (NE), 5-hydroxytryptamine (5-HT), and dopamine (DA) [34]. It has been reported that acupuncture's antidepressive effects may involve neuropeptide Y (NPY) activation in the hypothalamus [28]. One research has reported that the mechanism underlying the antidepressant-like effects of EA may associate with the enhancement of amplifying neural progenitors (ANPs) proliferation and preserving quiescent neural progenitors (QNPs) from apoptosis [35]. In our study, we found that EA may enhance p-ERK1/2 and p-p38 in the HP on depression rats. Lu et al. [8] have reported that acupuncture treatment could alleviate depressive-like behaviors, including OFT and sucrose intake, and its mechanism could activate ERK-CREB pathway. We got the same result that acupuncture improves the p-ERK1/2 in the hippocampus, despite the different results on OFT. We indicated that acupuncture could relieve some symptoms in this animal model of depression, including forced swimming test and sucrose solution intake. In that article, EA was compared to Paroxetine and both of them were equally effective. However, in our study, EA was compared to placebo control in order to make the effect of EA clear. Although EA could increase p-ERK1/2 in the hippocampus, which has been reported by some studies [8, 36], we found that EA could enhance the p-p38. The MAPK family is subdivided into three main classes: ERK, Jun N-terminal kinases (JNK), and the p38 kinase, and they all are involved in differentiation, survival, and structural and functional plasticity of neurons. So far, only ERK pathway was examined in animal model of depression and effect of acupuncture. However, the levels of JNK and p38 kinases can be also altered in depression [37]. In order to determine whether EA could play the role on p-p38 in hippocampus, we detected the level of p-p38 kinases and found that EA could improve the p-p38 and p-ERK1/2 in the hippocampus at the same time.

## 5. Conclusion

In conclusion, we found that EA could alleviate unpredictable chronic mild stress-induced depression-like behavior. EA could improve p-ERK1/2 and p-p38 in the HP in the rats exposed to chronic unpredicted mild stress. Our results suggest that the antidepressant-like effect of acupuncture might be mediated by modulating the p-ERK1/2 and p-p38MAPK pathway in the hippocampus. Further studies to investigate EA's mechanisms of action on depression are needed.

## Figures and Tables

**Table 1:** Unpredictable stressors for 14 days.

Day	Time of 1st stressor	1st stressor	Time of 2nd stressor	2nd stressor
1	10:00 a.m.	10°C swim stress (4 min)	4:00 p.m.	Lights off (2 h)
2	12:30 p.m.	Lights off (3 h)	7:00 p.m.	Lights on overnight
3	10:00 a.m.	4°C cold isolation (20 min)	4:00 p.m.	Cage shaking (5 min)
4	11:00 a.m.	4°C cold isolation (1 h)	2:00 p.m.	Restraint stress (40 min)
5	3:00 p.m.	Lights off (2 h)	5:00 p.m.	Cage tilting 45° (2 h)
6	10:00 a.m.	Food deprivation (24 h)		
7	10:00 a.m.	Water deprivation (24 h)		
8	3:30 p.m.	10°C swim stress (15 min)	7:00 p.m.	Lights on overnight
9	4:00 p.m.	Restraint stress (1 h)	5:00 p.m.	Lights off (1 h)
10	10:00 a.m.	Cage shaking (5 min)	3:30 p.m.	4°C cold isolation (15 min)
11	12:00 p.m.	Lights off (2 h)	7:00 p.m.	Lights on (2 h)
12	10:00 a.m.	Restraint stress (1 h)	2:00 p.m.	10°C swim stress (5 min)
13	10:00 a.m.	Food deprivation (24 h)		
14	10:00 a.m.	Water deprivation (24 h)		
